# A new species of spiny *Solanum* (Solanaceae) from Peru

**DOI:** 10.3897/phytokeys.39.7513

**Published:** 2014-06-20

**Authors:** Stephen R. Stern

**Affiliations:** 1 Department of Biological Sciences, Colorado Mesa University, 1260 Kennedy Ave, Grand Junction, CO 81501

**Keywords:** *Solanum*, Peru, new species, Torva clade

## Abstract

A new species of *Solanum* is described from Peru. *Solanum junctum* S. Stern & M. Nee, **sp nov.** is a member of the Torva clade of the spiny solanums (Leptostemonum clade). The narrow corolla lobes and recurved prickles of *Solanum junctum* are similar to species in the Micracantha clade, but *Solanum junctum* differs in its branched inflorescences and upright green fruits. These characteristics are shared with other members the Torva clade; within this section *Solanum junctum* is morphologically most similar to *Solanum subinerme* and *Solanum poinsettiifolium*. *Solanum subinerme* has larger flowers, longer cauline prickles, and often has long straight prickles on the adaxial leaf surface that are lacking in *Solanum junctum*. *Solanum poinsettiifolium* has fewer spines, dense white tomentum on the abaxial leaf surfaces, stout unbranched inflorescences, and more extensive interpetalar corolla tissue than *Solanum junctum*.

## Introduction

The giant genus *Solanum* L. has been the subject of recent systematic studies due to support from the NSF Planetary Biodiversity Inventory program, including phylogenetic study of the Leptostemonum clade (Bohs 2005; Levin et al. 2006; Weese and Bohs 2007; Stern et al. 2010; Stern et al. 2011). This clade includes approximately 350–400 species commonly known as the “spiny solanums” due to their epidermal prickles. Species in the group also have long, attenuate anthers and stellate hairs and exhibit a variety of habits, from small herbs to large trees.

Various species in the clade are vines or lianas that climb using recurved prickles. Recurved prickles are uncommon in *Solanum* and have been used as a synapomorphy to define the *Solanum lanceifolium* species group (Whalen 1984) also known as sect. *Micracantha* Dunal (Nee 1999). Stern et al. (2011) conducted a large-scale phylogenetic study of the spiny solanums and found that *Solanum* species that climb with recurved prickles belong to several different groups, including the Torva, Erythrotrichum, Micracantha, and Old World clades (as defined by Stern et al. 2011). Clearly, climbing via recurved prickles is a trait that has evolved multiple times in different lineages.

Revisionary work on the Micracantha clade and phylogenetic study of the large Torva clade has led to the identification of the new species described here. It has been widely collected throughout the central and northern Andes in Peru with specimens dating from the 1920’s. Macbride (1962) cited some of these in his treatment of *Solanum heterophyllum* Lam. in the Flora of Peru (now recognized as a synonym of *Solanum subinerme* Jacq., a species in the Torva clade). He noted that his concept of *Solanum heterophyllum* included specimens that were variable and indicated that the specimens cited “may be several species.” Indeed, these specimens represent at least three different *Solanum* species. Nee et al. (2006) used some of the material that Macbride called *Solanum heterophyllum*, including *Mexia 6485* and *Williams 3448*, to describe *Solanum pedemontanum* M. Nee, a species of the Micracantha clade. Another specimen Macbride included under *Solanum heterophyllum*, *Swingle 119*, is *Solanum poinsettiifolium* Rusby, a member of the Torva clade. Finally, other specimens Macbride cited, including *Williams 7678* and *Klug 3407*, are used to describe the new species below. Specimens of the new species have been annotated by Nee as a species “at junction of sect. *Micracantha* with sect. *Erythrotrichum* Whalen ex. A. Child.” but morphological and molecular data support its inclusion in the Torva clade.

## Taxonomy

### Solanum junctum

Taxon classificationPlantaeSolanalesSolanaceae

S. Stern & M. Nee sp. nov.

urn:lsid:ipni.org:names:77140259-1

Fig. 1

#### Type.

**Peru**. **Pasco**: Prov. Oxapampa, Dist. Pozuzo, 1 km N del Puente Yulitunqui–Sector Huampal, Parque Nacional Yanachaga-Chemillén, 10°09'47"S, 75°33'58"W, 975–1100 m, 15 Apr 2005 (fl), *E. Ortiz V. & J. Mateo M. 576* (holotype: NY[NY01802055]!; isotypes: AMAZ, F, HAO, HOXA, HUT, MO!, MOL, USM, NY[NY00854134]!, NY[NY01802056]!)

#### Diagnosis.

Similar to *Solanum subinerme* Jacq. and *Solanum poinsettiifolium* Rusby. *Solanum junctum* differs from *Solanum subinerme* in its smaller flowers, shorter cauline prickles and lack of straight prickles on the adaxial leaf surfaces. *Solanum junctum* has branched inflorescences and lacks the white tomentum on the adaxial leaf surface and the more abundant interpetalar tissue of *Solanum poinsettiifolium*.

Erect or scandent shrub, liana, or small tree, 1–20 m. Stems armed with recurved tan to brown prickles to 5 mm long, the base to 2–4 × 0.5–1 mm, the young stems moderately to densely pubescent with tan to white stellate hairs, the stalks absent to 1 mm, multiseriate, the rays 6–10, ca. 0.5 mm long, the midpoints absent to 0.5 mm, the older stems becoming sparsely pubescent to nearly glabrous. Flowering portions of stem of difoliate sympodial units, the leaves usually geminate, those of a pair often slightly unequal. Leaves simple, the blades 8–12 × 4–7 cm, ovate, chartaceous, green on both surfaces with the adaxial surface typically darker, both surfaces moderately pubescent with hairs like those of the stem, the abaxial surface typically slightly more tomentose and often armed with a few recurved prickles to 2 mm in length on the midrib, abaxial surface with occasional simple glandular hairs below the stellate hairs; venation pinnate, the secondary veins 3–6 on each side of the midvein; base rounded to obtuse, often asymmetrical; margin entire; apex acute to attenuate; petioles 1–3.5 cm, moderately pubescent with hairs like those of the stem, sparsely to moderately armed with prickles like those of the stem. Inflorescence 5–8 (10) cm, extra-axillary, branched with 2–3 (5) main branches, bearing 14–20 flowers, the plants apparently andromonoecious, with male flowers lacking a developed style and hermaphroditic flowers with an elongated style, the axes moderately to densely pubescent with hairs like those of the stem, unarmed; peduncle 2–10 mm; rachis 4–7 (10) cm; pedicels 4–10 mm in flower, 8–14 mm in fruit, spaced 1–3 mm apart, articulated at the base. Flowers 5-merous, appearing actinomorphic on herbarium sheets but slightly zygomorphic in the field due to curved anthers, the flower buds slightly curved. Calyx 0.5–3 mm long in bud through anthesis, cupular with lobes nearly absent, moderately to densely pubescent with hairs like those of the stem, the calyx splitting into lobes during late flowering or early fruiting; fruiting calyx lobes 2–3 × 1–2 mm, triangular. Corolla 2–3 cm in diameter, chartaceous, light violet to purple, lobed nearly to the base, the lobes 8–12 × 2–3.5 mm, narrowly triangular, densely pubescent on the abaxial surface with hairs like those of the stem, glabrous to sparsely stellate-pubescent on the midvein on the adaxial surface. Stamens 8-12 mm long; filaments ca. 0.5 mm long, glabrous; anthers 8–12 × 1–1.5 mm, proximally curving downward with small upward curve at distal end, attenuate, tapering, connivent to weakly spreading, yellow, the base cordate, the apex obtuse, the pores apical, not opening into longitudinal slits with age. Ovary glabrous; style in functionally male flowers 1–2 × ca. 0.5 mm, style in hermaphroditic flowers 12–14 × 0.5 mm, exceeding the anthers by 2–3 mm, cylindrical, glabrous; stigma ca. 0.5 mm wide. Fruit a globose berry 1–1.2 cm in diameter, green, glabrous, held upright, with 3–10 fruits per infructescence. Seeds 75-120 per fruit, 3–4 × 2–3 mm, ovate to reniform, brown.

**Figure 1.**
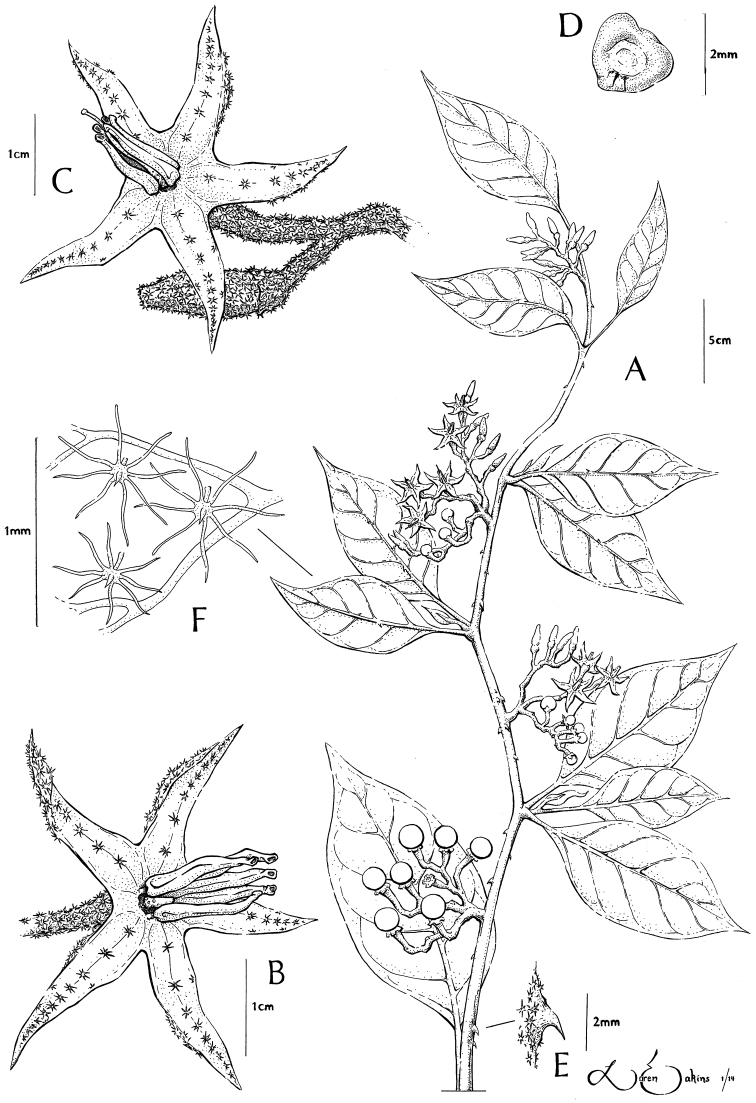
*Solanum junctum* S. Stern & M. Nee **A** Habit **B** Functionally male flower; note reduced style **C** Perfect flower **D** Seed **E** Cauline prickle **F** Trichomes on abaxial leaf surface. All from *Ortiz & Mateo 576*.

#### Distribution and phenology.

Known from Amazonas, Ayacucho, Junín, Pasco, and San Martín Departments in Peru from 600–1800 m in elevation. Flowering specimens were collected in February-May, July, November-December and fruiting specimens in April, July-August, November.

#### Etymology.

*Solanum junctum* is taken from the Latin “junctus“ for “connect or join,” referring to the morphological similarities of this species with other sections within the spiny solanums. This has been used as an herbarium name on specimen annotations by M. Nee since at least 1995.

#### Conservation status.

The conservation status of *Solanum junctum*, according to the IUCN Red List Categories (IUCN, 2010) is Least Concern due to the large extent of occurrence (~99,000 km^2^) and numerous collections (Bachman et al. 2011).

Since *Solanum* is such a large and diverse group, particularly in the Andes, it is not surprising that many new species and taxonomic difficulties remain. This is particularly true in undercollected areas of the Andes, but recently many inroads have been made (Knapp 2010; Stern and Bohs 2010; Farruggia and Bohs 2010; Tepe et al. 2012; Särkinen et al. 2013). Field collections, revisionary, and phylogenetic study have spurred the description of species that otherwise may remain as “herbarium names”.

Both M. Nee and S. Stern recognized *Solanum junctum* as a new species from herbarium collections, resulting in the shared authorship of this name. Stern first annotated a specimen as a new species in 2008 (*Rodríguez & Leiva 2121* HAO, subsequently destroyed in an herbarium fire) and Nee has applied the herbarium name *Solanum junctum* to specimens since at least 1995 (*Schunke 6020* [NY]). The type material for *Solanum junctum* was selected from the numerous collections made from Prov. Oxapampa in the Department of Pasco, Peru. *Ortiz & Mateo 576* was chosen as the type due to the quality of the specimens and wide distribution in herbaria. The holotype at NY was the highest quality specimen of those seen and contained abundant flowering material and developing fruits.

As with many *Solanum* species with recurved prickles, *Solanum junctum* has a variable habit and may be an erect or scandent shrub, vine, or even a small tree. The species is unusual in the Torva clade in having flowers with very narrow corolla lobes with sparse interpetalar tissue, but its branched inflorescences and green upright fruits are shared with many other species in this section. DNA sequence data from specimens of *Solanum junctum* have been added to the dataset of Stern et al. (2011) and indicates that *Solanum junctum* is a member of the Torva clade but the exact species level relationships remain unclear. Phylogenetic relationships between members of this group are being assessed further using molecular data (S. Stern, in prep.).

Morphologically, the violet to purple flowers with narrow corolla lobes of *Solanum junctum* are similar to those of *Solanum subinerme*. Additionally, both species have curved flower buds and slightly zygomorphic flowers due to curved anthers. These species can be differentiated by the larger flowers of *Solanum subinerme*, with corollas 3.5–4 cm in diameter (versus 2-3 cm in diameter in *Solanum junctum*), the longer cauline prickles in *Solanum subinerme*, and presence of long straight prickles on the adaxial leaf surface in *Solanum subinerme*, which are lacking in *Solanum junctum*. Finally, *Solanum subinerme* has thin pedicels that reach 2 cm or more in fruit while those of *Solanum junctum* are thicker and only reach 14 mm. *Solanum subinerme* has a much broader distribution and is found from the Caribbean through northern South America to the Amazon Basin. It is not known from the higher elevations of Peru where *Solanum junctum* is found.

*Solanum junctum* is also similar to *Solanum poinsettiifolium*, a species ranging from Dept. Beni, Bolivia along the eastern slope of the Andes to central Peru. *Solanum poinsettiifolium* is represented by numerous collections from the area where Ucayali, Huánuco, and Loreto Departments intersect. These superficially resemble *Solanum junctum* as they share similar leaf morphology, flowers and fruits that are a similar size and color, and both species have curved flower buds. These species differ in that *Solanum poinsettiifolium* specimens are all described as trees or shrubs, have very few spines on the stem and none on the abaxial leaf surface, and have dense white tomentum with a soft, velvety appearance on the abaxial leaf surfaces. The corolla of *Solanum poinsettiifolium* has more abundant interpetalar tissue and the inflorescence is stout and unbranched.

Some specimens of *Solanum ovalifolium* Dunal (another member of the Torva clade) may also resemble *Solanum junctum*. *Solanum ovalifolium* is a shrub to small tree with much smaller flowers than *Solanum junctum* (corollas typically under 1 cm in diameter in *Solanum ovalifolium* vs. 2–3 cm in diameter in *Solanum junctum*). The inflorescences of *Solanum ovalifolium* are generally larger and more branched than those of *Solanum junctum* and may branch further up the rachis, whereas the inflorescences in *Solanum junctum* branch very near the base. *Solanum ovalifolium* ranges from Venezuela, Colombia, and Ecuador to Depts. Amazonas and Cajamarca in northern Peru, where its distribution appears to terminate at the Amotape-Huancabamba zone (Stern and Bohs 2010).

It is also possible to confuse *Solanum junctum* with *Solanum pedemontanum*, a member of the Micracantha clade. At least two specimens of *Solanum pedemontanum* (*Krukoff 8421* and *McDaniel & Rimachi 16879* at NY) have been annotated as possible *Solanum junctum* by M. Nee. Macbride (1962) cited specimens belonging to both *Solanum junctum* and *Solanum pedemontanum* in his taxonomic treatment of *Solanum heterophyllum*. While the habit of *Solanum junctum* ranges from a vine to shrub, *Solanum pedemontanum* is nearly always described as a scrambling vine. In Peru, *Solanum pedemontanum* tends to occur at lower elevations (100-450 m) than *Solanum junctum* (600–1800 m). The corolla in *Solanum pedemontanum* is white versus the light purple corolla of *Solanum junctum* and the corolla lobes of *Solanum pedemontanum* are slightly longer (12–20 mm) than those of *Solanum junctum* (8-12 mm). The inflorescence in *Solanum pedemontanum* is unbranched, whereas it branches at the base in *Solanum junctum*. Finally, fruits in *Solanum pedemontanum* are orange to red whereas they remain green at maturity in *Solanum junctum*.

#### Paratypes.

Peru. **Amazonas:** Prov. Condorcanqui, Distrito El Cenepa, Región NE del Marañón, Puerto Mori, Río Comaina, 4°23'S, 78°21'W, 800 m, 19 Aug 1994 (fr) *R. Vásquez et al. 18921* (BM, MO, NY, USM); Bagua Dist., Aramango, Cerros de Nueva Esperanza, 5°28'02"S, 78°23'11"W, 1800 m, 20 Dec 2001 (fl), *R. Vásquez et al. 27499* (NY, USM). **Ayacucho:** Prov. La Mar, alrededores de Buena Gana, aprox. 8.5 km lineales al WNW de San Antonio, Dist. Anco, 1775 m, 21 Apr 2007 (fl) *J. Roque 5475* (USM). **Cajamarca**: Prov. San Ignacio, Dist. San José de Lourdes, Caserío Estrella del Oriente, 4°50'S, 78°55'W, 1600–1650 m, 14 Nov 1998 (fl), *E. Rodríguez R. & S. Leiva G. 2121* (HAO [destroyed], HUT). **Junín**: Track to Chipita, 11°07'70"S, 75°21'19"W, 1400 m, 16 Nov 2002 (fl, fr) *Monro, Pennington, & Diaz 4005* (BM); Chanchamayo Valley, Mar. 1924–1927 (fl), *Schunke 264* (F, US). **Pasco**: Prov. Oxapampa, Dist. Pozuzo, Sector Huampal, 10°10'45"S, 75°34'26"W, 1000–1200 m, 20 Jul 2006 (fl, fr), *Cárdenas & Flores 578* (NY); Dist. Villa Rica, Comunidad Nativa San Pedro de Pichanaz, sector San Francisco, 10°26'24"S, 75°26'07"W, 600 m 17 Apr 2007 (fl) *M. Huamán & R. Rivera 174* (NY); Oxapampa, Dist. Pozuzo, Parque Nacional Yanachaga Chemillén Pozuzo Sector Pan de Azúcar, Zona de Recuperación, 10°15'S, 75°13'W, 1100–1250 m, 10 Apr 2003 (fl, fr), *A. Monteagudo et al. 4912* (NY); Oxapampa, Dist. Villa Rica, Cerro El Ascensor, Bosque de Protección San Matías–San Carlos, 10°45'S, 74°55'W, 1355 m, 3 Jul 2003 (fl), *J. Perea & C. Mateo 137* (NY); Prov. Oxapampa, Dist. Iscosaizín, carretera Chatarra–Puerto Bermúdez, 10°30'25"S, 75°04'06"W, 650 m, 23 Nov 2007 (fl, fr), *Tepe et al. 2264* (NY). **San Martín:** Zepelacio, near Moyobamba, 1200–1600 m, Dec 1933 (fl), *G. Klug 3407* (F, MO, US, WIS); Prov. Lamas, Alonso de Alvarado, carretera a Moyobamba, 800 m, 23 Apr 1973 (fl), *J. Schunke V. 6020* (NY). Prov. Lamas, Dist. Alonso de Alvarado, San Juan de Pacaizapa, km 72 carretera Tarapoto–Moyobamba, 1000–1050 m, 29 May 1977 (fl), *J. Schunke 9536* (MO, NY); San Roque, 1350–1500 m, 3 Feb 1930 (fl), *Ll. Williams 7678* (F, US).

## Supplementary Material

XML Treatment for Solanum junctum
